# Decrease in Seminal HIV-1 RNA Load After Praziquantel Treatment of Urogenital Schistosomiasis Coinfection in HIV-Positive Men—An Observational Study

**DOI:** 10.1093/ofid/ofx199

**Published:** 2017-09-15

**Authors:** Nicholas Midzi, Takafira Mduluza, Boniface Mudenge, Leslie Foldager, Peter D C Leutscher

**Affiliations:** 1 Department of Medical Microbiology, College of Health Sciences, University of Zimbabwe, Avondale, Harare, Zimbabwe; 2 Department of Biochemistry, University of Zimbabwe, Harare, Zimbabwe; 3 School of Laboratory Medicine and Medical Sciences, University of KwaZulu Natal, Durban, South Africa; 4 Flow Cytometry Centre, Harare, Zimbabwe; 5 Department of Animal Science, Aarhus University, Aarhus, Denmark; 6 Bioinformatics Research Centre, Aarhus University, Tjele, Denmark; 7 Centre of Clinical Research, Regional Hospital North Denmark, Denmark; 8 Clinical Institute, Aalborg University, Denmark

**Keywords:** HIV, HIV-1 RNA, *Schistosoma haematobium*, semen, praziquantel, urogenital schistosomiasis

## Abstract

**Background:**

Urogenital schistosomiasis due to *Schistosoma hematobium* infection is hypothesized to cause increased HIV-1 RNA shedding in semen in HIV co-infected men as result of chronic egg-induced inflammation in the prostate and the seminal vesicles. The effect of treatment with the antihelminthic agent praziquantel on seminal HIV-1 RNA load was assessed in this study.

**Methods:**

HIV-1 RNA load was determined in blood plasma and semen at baseline and at 10-week follow-up. Praziquantel was administered at baseline and two weeks later.

**Results:**

Eighteen HIV-positive men with *S. haematobium* co-infection were enrolled into the study. Status of antiretroviral therapy (ART): 6 ART-naïve and 12 ART-experienced. All participants became egg-negative in urine at follow-up. Among the ART-naïve men, the mean HIV-1 RNA load decreased by 0.32 log_10_ copies per mL (4.41 vs 4.09) in blood plasma from baseline to follow-up, and in semen by 1.06 log_10_ copies per mL (4.06 vs 3.00).

**Conclusions:**

This study demonstrated a decline in seminal HIV-1 RNA load following praziquantel treatment of urogenital schistosomiasis infection in HIV-positive men. The finding needs further exploration in a larger randomized study targeting praziquantel as a supplementary preventive measure of sexual transmission of HIV-1 in *S. haematobium* endemic areas in sub-Saharan Africa.

Urogenital schistosomiasis is caused by infection with the helminth parasite *Schistosoma haematobium* [1]. Transmission occurs when larval cercariae in the fresh water bodies penetrate the skin. Subsequently, the adult worm pairs migrate to the venous plexus of the pelvic organs, where eggs are produced for many years if the host is left without treatment. A proportion of those eggs are sequestered, giving rise to establishment of chronic inflammatory granulomatous lesions in the urinary bladder and the internal genitals in males and females living in *S. haematobium* endemic areas. Estimates show that about 140 million people are infected with *S. haematobium* [2]. About 85% of those live in sub-Saharan Africa, where the distribution overlaps with areas of high HIV-1 prevalence [3, 4].

The World Health Organization (WHO) has urged for increased research awareness toward the role of genital schistosomiasis in HIV transmission in sub-Saharan Africa [5]. Community-based studies from Zimbabwe, Tanzania, and Mozambique have consistently found increased HIV prevalence among women with female genital schistosomiasis (FGS) [6–8]. Presence of chronic egg-induced inflammatory lesions and increased vascularity as part of the FGS pathology is considered an underlying mechanism involved in facilitating entry of the HIV-1 RNA virus across the friable mucosa in the cervix and the vagina [9–11]. Presence of high densities of CD4+ T lymphocytes and macrophages surrounding schistosome eggs in the subepithelial tissue of the cervix may also contribute to the increased susceptibility to HIV infection [12].

Male genital schistosomiasis (MGS) has been hypothesized to constitute a risk factor for HIV transmission through increased HIV-1 RNA shedding in semen [13] in a similar manner as observed in men with gonococcal urethritis [14]. Postmortem studies have shown that the seminal vesicles and the prostate are affected by the egg-induced inflammation as frequently as the urinary bladder [15–17]. In support of the hypothesis, MGS has been found to be associated with leucocytospermia and increased seminal levels of pro-inflammatory cytokines [18, 19]. Those abnormalities tend to normalize after treatment with praziquantel, which takes effect by killing the adult worms, followed by resolution of the egg-induced inflammation of the affected urogenital organs within a few weeks [20]. In the HIV co-infected with urogenital schistosomiasis person, presence of HIV-1 hosting immune cells in the seminal vesicles and the prostate is expected to decline after praziquantel treatment, with diminished viral shedding in semen as a result.

To date, no studies have been conducted to clarify the impact of MGS on HIV transmission and the role of praziquantel treatment as a potential measure in the overall HIV control strategy in *S. haematobium* endemic areas in sub-Saharan Africa.

We conducted this study to assess the potential effect of praziquantel treatment on HIV-1 RNA load in blood plasma and semen in Zimbabwean HIV-positive men co-infected with urogenital schistosomiasis.

## METHODS

### Study Design and Area

The study design was a prospective, sequential comparison of 2 cohorts of HIV and *S. haematobium* co-infected men. The study was conducted in Ngundu area (Chivi district), Masvingo province in Zimbabwe from April to October 2015. Selection of the site was based on results from the recent national schistosomiasis survey that showed high prevalence of urogenital schistosomiasis among primary school–age children in the Ngundu area [21].

### Study Participants

**Figure 1.**
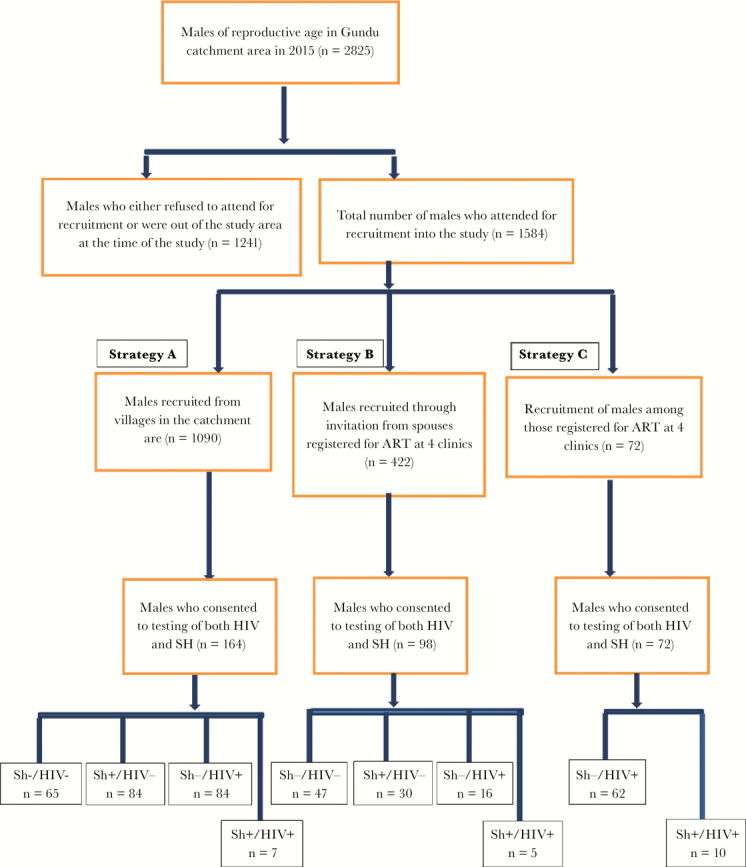
Flow chart showing the population of males of reproductive age in the Ngundu area and how these males were approached by different inclusion strategies (A, B, and C, respectively), leading to identification of 22 eligible candidates co-infected with HIV and schistosomiasis, among whom 18 took part in the final study. Abbreviations: ART, antiretroviral therapy; SH, *Schistosoma haematobium*.

HIV-positive men age 18 to 49 years were recruited from the catchment area (population 21 000) of Ngundu Rural Health Centre, which serves as an opportunistic infection (OI) clinic among other essential health services for the community. The population of males of reproductive age (15–49 years) in the catchment area was 2825 (Figure 1). Of these, 1584 (78.6%) males accepted an invitation to the study. As the main aim was to screen as many males as possible in the community in order to identify males coinfected with urogenital schistosomiasis and HIV-1, the first strategy (A) involved screening of *S. haematobium* on day 1 among all males willing to join the study. Men who tested positive for *S. haematobium* were invited to Ngundu Rural Health Centre for a further informed consent form administration if they were willing to join the clinical studies, for which another urine sample would be collected on 2 successive days. Also, semen and blood would then be donated. This rather costly strategy (A) recruited 1090 males, but the yield of males coinfected with urogenital schistosomiasis and HIV-1 (n = 7; 0.6%) was discouragingly low (Figure 1). Thus, a second strategy B and, subsequently, a third strategy C were devised. In strategy B, women registered for antiretroviral therapy (ART) at 4 health facilities in the catchment area were asked to invite their male spouses to the study. The urine screening and recruitment procedure were repeated as in strategy A. Among 422 males invited, only 98 were recruited, and of those 5 (5.1%) were co-infected with *S. haematobium* and HIV. The third strategy C involved invitation of males registered for ART at the 4 health facilities within the catchment area. From the 72 HIV-positive males recruited, 10 (13.9%) participants were coinfected with *S. haematobium*. Finally, 22 males co-infected with *S. haematobium* and HIV-1 identified using the 3 strategies (A+B+C) were considered for the pilot study. Of those, 4 failed to donate semen and were excluded from the study. The remaining 18 participants were classified in accordance with antiretroviral therapy status: ART-naïve participants and ART-experienced participants on consistent ART in the last 6 months prior to enrollment in the study. ART-experienced participants were being treated with the following regimens: tenofovir + lamivudine and nevirapine *or* tenofovir + lamivudine and lopinavir/ritonavir.

### Study Procedures

Screening for *S. haematobum* infection was performed by examination of urine samples collected on 3 consecutive days from each subject using the urine filtration technique [22]. Intensity of *S. haematobium* infection in each participant was determined by the mean egg count observed in 3 successive days: mild (1–9 eggs/10 mL urine), moderate (10–49 eggs/10 mL urine), and heavy (≥ 50 eggs/10 mL urine). A stool sample from each participant was examined for presence of *S. mansoni* eggs and soil-transmitted helminths by the whole stool formal ether concentration technique.

The measurement of CD4+ cell count was conducted in 5 mL of whole blood in a BD Vacutainer blood collection tube for CD4 stabilization, inverted 8 to 10 times, and stored at ambient temperature (2–30^o^ C) prior to analysis by flow cytometry.

For measurement of HIV-1 viral load in blood, 5 mL of whole blood was collected using an EDTA tube. The specimen was centrifuged for 5 minutes at 3000 rpm to obtain plasma, 0.5 mL of which was then added to a NucliSENS Lysis Buffer tube, vortexed thoroughly, and stored at 2–8^o^C prior to HIV-1 RNA analysis. Semen samples were ejaculated into a medical condom (*Male-FaktorPak*) or a sterile container according to preference of the participant. The semen was allowed to liquefy for 1 hour at room temperature. The specimen was then centrifuged for 5 minutes at 5000 rpm; then 0.2 mL of the seminal plasma was added to the NucliSENS Lysis Buffer tube provided, vortexed thoroughly, and stored at 2–8^o^C. HIV-1 RNA analyses were carried out within 24 hours after receipt of blood and semen samples. NucliSENSEasyQ HIV-1 v2.0 assay was used for HIV-1 RNA quantification in blood and seminal plasma. The lower detection limit of the HIV-1 RNA concentration was 20 copies/mL (log_10_ 1.3 copies/mL). The result of the HIV-1 measurement in blood and seminal plasma was categorized as quantifiable (≥20 copies/mL); detectable, but not quantifiable (<20 copies/mL); or nondetectable.

Sexually transmitted infection (STI) agents (*Neisseria gonorrheae*, *Chlamydia trachomatis*, *Mycoplasma genitalium*, and/or *Trichomonas vaginalis*) were assessed by the Nucliscense easyQ polymerase chain reaction (PCR) machine using the Nuclic acid sequencing base assay (Nasba) PCR real-time technique.

Participants were assigned treatment with PZQ (40 mg/kg bodyweight) at closure of the baseline study and 2 weeks later to increase efficacy of therapy.

### Study Outcome Measures

The primary outcome measure was HIV-1 RNA viral load (log_10_ copies/mL) in plasma and in semen at follow-up in comparison with viral load at baseline prior to praziquantel treatment. Other outcome measures were urinary egg count (ova/10 mL urine) and CD4+ cell count (cells/mm^3^).

### Ethical Consideration

The study was approved by the national ethical review board, Medical Research Council of Zimbabwe (MRCZ Ref: MRCZ/A/1853), and the community leadership (Provincial Medical Director, District Administrator, chiefs, village headmen, and councilors), and by the participants following signing of the informed consent.

### Statistical Analysis

**Figure 2.**
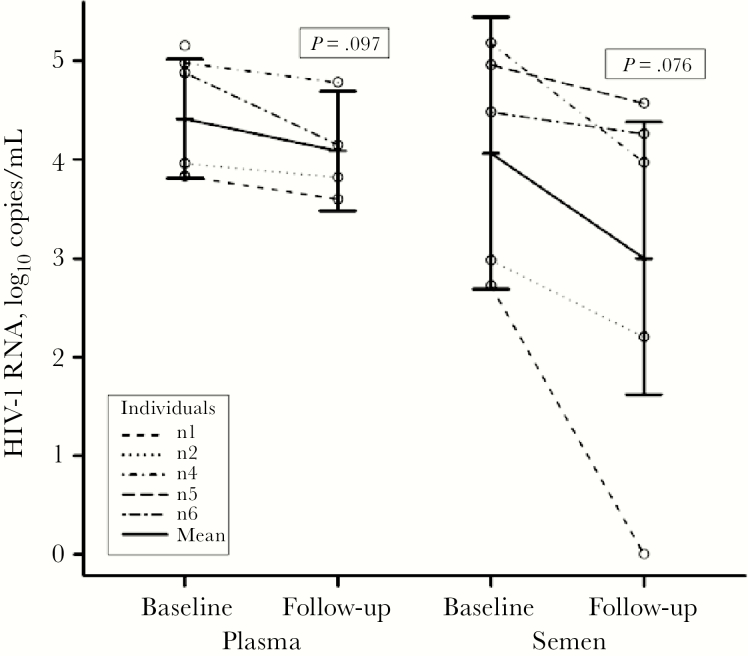
Comparison of paired HIV-1 RNA loads in plasma and in semen, respectively, in antiretroviral therapy (ART)–naïve patients at baseline and at follow-up after praziquantel treatment. SPSS version 16 and R version 3.2.1 were used for data analysis [23]. *S. haematobium* infection intensity was expressed as number of eggs/10 mL urine. Mean blood plasma and semen HIV-1 RNA copies counted were log_10_ transformed. Calculation of mean HIV-1 RNA load in plasma, or an HIV-1 RNA result of less <1.3 log_10_ copies in semen, was determined to be 1.28 log_10_ copies (19 copies)/mL, and a negative result to be 0 log_10_ copies /mL. A paired *t* test was applied to test for changes in mean HIV-1 RNA load from baseline to follow-up. The confidence intervals in Figure 2 were obtained by use of a linear mixed model with random intercept.

SPSS version 16 and R version 3.2.1 were used for data analysis [23]. *S. haematobium* infection intensity was expressed as number of eggs/10 mL urine. Mean blood plasma and semen HIV-1 RNA copies counted were log_10_ transformed_._ Calculation of the mean HIV-1 RNA load in plasma, or an HIV-1 RNA result of less <1.3 log_10_ copies in semen, was determined to be 1.28 log_10_ copies (19 copies)/mL and a negative result to be 0 log_10_ copies/mL. A paired *t* test was applied to test for changes in mean HIV-1 RNA load from baseline to follow-up. The confidence intervals in Figure 2 were obtained by use of a linear mixed model with random intercept.

## RESULTS

### Baseline

Among the 18 study participants with HIV-1 and urogenital schistosomiasis co-infection, 6 were ART naïve and 12 ART experienced. The median age of the participants was 34 years (range, 29*–*38 years) in ART-naïve group and 35 years (range, 21*–*49 years) in the ART-experienced group (Table 1). Five of the 6 ART-naïve participants were found with moderate *S. haematobium* infection intensity, and 1 participant had mild infection intensity, whereas the distribution of infection intensity in the ART-experienced group was as follows: heavy (n = 2), moderate (n = 3), and mild (n = 7). None of the 18 participants were identified with *S. mansoni* eggs in feces, and they were all diagnosed negative for STI by PCR.

**Table 1.** Urinary Egg Count, CD4 Count, and HIV-1 RNA Load in HIV-Positive Men Co-infected With Urogenital Schistosomiasis in Accordance With Antiretroviral Therapy Status: Naïve vs Experienced

Individuals	Age					HIV-1 RNA, log10 copies/mL^a^			
		Intensity of *Schistosoma haematobium* Infection^b^		CD4+ Count, cells/mm^3^		Plasma		Semen	
		Baseline	Follow-up	Baseline	Follow-up	Baseline	Follow-up	Baseline	Follow-up
ART-naïve (n = 6)									
n1	38	Moderate	Negative	382	301	3.83	3.60	2.72	ND
n2	32	Mild	Negative	412	621	3.96	3.82	2.98	2.20
n3	29	Moderate	NA	340	NA	4.80	NA	1.94	NA
n4	36	Moderate	Negative	237	255	4.98	4.78	5.18	3.97
n5	34	Moderate	Negative	35	NA	5.15	NA	4.96	4.57
n6	34	Moderate	Negative	172	176	4.88	4.15	4.48	4.26
ART-experienced (n = 12)									
e1	21	Mild	Negative	230	311	<1.3	ND	<1.3	ND
e2	43	Mild	Negative	316	343	<1.3	ND	<1.3	NA
e3	49	Mild	Negative	222	164	2.14	1.83	<1.3	ND
e4	23	Mild	Negative	509	468	<1.3	ND	<1.3	<1.3
e5	36	Mild	Negative	251	246	<1.3	<1.3	<1.3	<1.3
e6	42	Mild	Negative	521	660	<1.3	ND	<1.3	ND
e7	33	Mild	Negative	359	474	<1.3	<1.3	<1.3	ND
e8	45	Moderate	Negative	348	249	ND	ND	ND	2.0
e9	37	Moderate	Negative	76	114	2.65	2.43	3.20	ND
e10	29	Moderate	NA	378	NA	<1.3	NA	3.20	NA
e11	33	Heavy	Negative	625	395	2.11	1.38	ND	ND
e12	24	Heavy	Negative	631	468	ND	NA	ND	ND

Abbreviations: ART, antiretroviral therapy; NA, nonavailable; ND, nondetectable.

^a^Lower detection limit was 1.3 log_10_ copies (20 copies)/mL.

^b^Intensity of *Schistosoma haematobium* infection by urine egg count (eggs/10 mL urine): mild (1–9), moderate (10–49), and heavy (≥50).

The mean CD4 count was 263 cells/ mm^3^ (range, 35–412 cells/mm^3^) in the ART-naïve group in comparison with 372 cells/mm^3^ (range, 76–631 cells/mm^3^) in the ART-experienced group (Table 1). Two participants in the ART-naïve group were observed with a CD4 count of less than 200 cells/mm^3^.

In the ART-naïve group, the mean blood plasma viral load was 4.59 log_10_ copies per mL (range, 3.83–5.15 log_10_ copies per mL) whereas mean seminal viral load was 3.71 log_10_ copies per mL (range, 1.94–5.18 log_10_ cps/mL). In the ART-experienced group, the mean plasma viral load was 1.31 log_10_ copies per mL (range, 0–2.65 log_10_ copies per mL), with the following distribution of viral load categories: negative (n = 2); <1.3 log_10_ copies/mL (n = 7) and ≥1.3 log_10_ copies/mL (n = 3). The mean seminal viral load was 1.28 log_10_ copies per mL (range, 0–3.20 log_10_ copies per mL), with the following distribution of viral load categories: negative (n = 3); <1.3 log_10_ copies/mL (n = 7) and ≥1.3 log_10_ copies/mL (n = 2). Of interest, in 2 ART-experienced participants (ID e9 and e10, respectively), the seminal viral load was 3.20 log_10_ copies per mL, which was a higher concentration than measured in blood plasma (Table 1).

### Follow-up

One of the 6 ART-naïve participants and 1 of the 12 ART-experienced participants were absent at follow-up. No changes in ART status had occurred for any of the remaining 16 participants during the follow-up period, and none of them were detected with eggs in urine or in feces. STI PCR in urine was persistently negative for all participants.

No statistical difference in mean CD4+ count was observed in the 2 study groups when comparing baseline with follow-up results. For the 4 ART-naïve participants, the mean CD4+ count was 301 cells/mm^3^ at baseline vs 338 cells/mm^3^ at follow-up. For the 11 ART-experienced participants, the mean CD4+ count was 372 cells/mm^3^ at baseline vs 354 cells/mm^3^ at follow-up survey.

The results of HIV-1 RNA measurement in plasma were available in 4 of the 5 ART-naïve participants present at follow-up, whereas results in semen were available in all 5 participants (Table1). Figure 2 shows plasma and seminal HIV-1 RNA concentrations at baseline and at follow-up in the ART-naïve participants. The mean plasma viral load in ART-naïve participants decreased by 0.32 (CI, –0.76 to 0.11) log_10_ copies per mL from 4.41 log_10_ copies per mL (range, 3.83–4.98 log_10_ copies per mL) at baseline to 4.09 log_10_ copies per mL (range, 3.60–4.78 log_10_ copies per mL) at follow-up up (*P* = .097). The mean seminal viral load decreased by 1.06 (CI, –2.31 to 0.18) log_10_ copies per mL, from 4.06 log_10_ copies per mL (range, 2.72–5.18 log_10_ copies per mL) at baseline to 3.00 log_10_ copies per mL (range, 0–4.57 log_10_ copies per mL) at follow-up (*P* = .076).

The results of HIV-1 RNA measurement in both blood plasma and in semen were available in 9 ART-experienced participants at follow-up (Table 1). Among the 3 participants observed with detectable HIV-1 RNA in plasma above the lower detection limit (1.3 log_10_ copies/mL) at baseline, the mean viral load decreased by 0.42 (CI, –1.10 to 0.26) log_10_ copies per mL from 2.30 log_10_ copies per mL (range, 2.11–2.65 log_10_ copies per mL) at baseline to 1.88 log_10_ copies per mL (range, 1.38–2.43 log_10_ copies per mL) at follow-up (*P* = .12). In the remaining 6 ART-experienced participant, 1 was observed with nondetectable viremia at baseline vs 4 participants at follow-up. Two of the 9 ART-experienced participants with complete paired HIV-1 RNA measurement results were found with a nondetectable viral load in semen at baseline, as opposed to 6 at follow-up.

## DISCUSSION

Despite the epidemiological overlap of urogenital schistosomiasis with HIV in sub-Saharan Africa [3, 4], no studies have been conducted to determine the effects of antischistosomal treatment with praziquantel on seminal HIV-1 RNA shedding in males co-infected with urogenital schistosomiasis. Literature on FGS and its public health implication, including its association with HIV transmission, is increasing [6, 7, 10, 24, 25], but only 2 community-based studies have generated data on MGS [13, 18, 19]. However, those studies did not seek to determine the role of MGS on HIV-1 transmission. Thus, to our knowledge, this is a first community-based scientific study reporting the results of the male genital schistosomiasis and HIV-1 in which the effect of praziquantel treatment of urogenital schistosomiasis on seminal HIV-1 RNA shedding was assessed.

The study has some limitations requiring cautious interpretation of the results due to (1) the small number of study participants, (2) the absence of a control group of HIV-positive men without urogenital schistosomiasis, and (3) the lack of diagnostic inflammatory markers in semen to support the study outcome.

In this setting, males were less forthcoming regarding visiting the health facilities for voluntary HIV counselling and testing (VCT). In general, males tend to seek health care late (to bed added) when HIV/AIDS is at advanced stage clinically. Some prefer to visit health facilities far away from their catchment areas in order to conceal their HIV status from family members including the wife. This made it more difficult for this study to yield a large number of HIV cases co-infected with urogenital schistosomiasis registered at local health facilities. On the other hand, cultural sensitivity toward donating semen by males and the voluntary nature of the study that allowed males to drop out of the study at any time imposed challenges. Several males opted out of the study during administration of informed consent form, which sought their autonomous decision to join the study following a full explanation about the study aims and methodology, which specified samples required from study participants.

It was shown in this study that praziquantel treatment of urogenital schistosomiasis in HIV co-infected men reduced semen viral load in all ART-naïve participants, but also to a minor extent in ART-experienced participants, a higher proportion of whom became HIV-1 RNA undetectable at follow-up in comparison with baseline. In addition, the seminal viral load of 3.20 log_10_ copies/mL was higher than the plasma viral load in 2 ART-experienced participants, even in 1 of them with a plasma viral load below the lower detection limit (<1.3 log_10_ copies/mL). Those findings may support the assumption that the seminal fluid–producing organs, primarily the prostate and the seminal vesicles, constitute a separate compartment containing an accumulated number of HIV-hosting immune cells as part of the egg-induced inflammatory environment in the pelvic organs. As a result, augmented numbers of HIV-1 RNA copies are shed into semen, presumably even when viral suppression in the blood is achieved by otherwise successful ART. In general, effective ART with an undetectable plasma viral load tends to limit the effect of urethritis on semen viral load, whereas when plasma viral load is poorly controlled, a high seminal viral load occurs, particularly in association with gonococcal urethritis [26]. However, the HIV RNA level in blood plasma is not reliable as an independent predictor of virus levels in semen, and the male genital tract is considered a distinct virological compartment from blood [27, 28]. Politch and colleagues have reported that STI and genital inflammation can override the suppressive effect of ART in seminal HIV shedding in HIV-infected men [29].

The ART-naïve participants in the study were diagnosed with light to moderate intensity of *S. haematobium* infection, defined as an egg count of 1 to 49 eggs/10 mL urine. The HIV-1 RNA load in semen may have been measured at an even higher level in ART-naïve participants with heavy intensity of infection (≥50 eggs/10 mL urine) due to an anticipated proportionally higher level of egg-induced inflammation in the urogenital organs, which contain a higher number in HIV-hosting immune cells, in concordance with findings in postmortem studies of *S. haematobium*–associated pathology in males [15, 16].

In a study by Dyer and colleagues, it was shown that HIV-1 RNA concentrations in blood and semen were significantly higher in HIV-positive men living in Malawi in comparison with US and Swiss HIV-positive men [30]. In a matched analysis of CD4+ cell counts between the 2 groups, a significant difference was also observed, with lower CD4+ cell counts in Malawian men. The findings may explain the high rates of HIV-1 sexual transmission and accelerated HIV-1 disease progression in sub-Saharan Africa. For further reflection, in Malawi, like in many countries in the southern Africa, schistosomiasis and other neglected tropical diseases are endemic, and there is growing evidence that schistosomiasis adversely impacts every phase of HIV/AIDS in co-infected individuals through inexpedient immune modulation [4, 31]. Individuals with *Schistosoma* have been found with higher cell surface densities of CCR5 and CXCR4 receptors on CD4+ T cells and monocytes in peripheral blood, which makes them more susceptible to HIV infection [32, 33].

Meta-analysis has shown that urethral infections due to STI pathogens are associated with significant increases in leukocyte concentrations and HIV-1 shedding in the genital tract [34]. These infections are likely to be particularly important in promoting the sexual transmission of HIV-1, and should therefore be the focus of HIV prevention strategies. Cohen and colleagues have shown that gonococcal and chlamydial urethritis in ART-naïve HIV-positive men in Malawi was associated with an increase in HIV-1 RNA concentrations in semen, which decreased significantly following treatment, in particular for gonococcal urethritis [14]. Similar observations have been made in ART-naïve HIV-positive men in a developed world setting being treated for urethritis, but not at the same significance level as the study in Malawi [35].

Urogenital schistosomiasis co-infection in HIV-positive men may constitute a neglected risk factor for HIV propagation in *S. haematobium* endemic areas in Africa due to increased HIV-1 RNA shedding in semen in an analogous way to the estimations in the probabilistic empiric model on high viral semen burden from inflammation in sub-Saharan Africa developed by Chakraborty and colleagues [36]. Because children and adults of both genders in those areas in most cases are chronically infected, the urogenital lesions therefore also tend to persist for decades, as opposed to lesions due to STIs, which are normally transient, with a few weeks’ duration in the clinical presentation. Hence, the contribution of urogenital schistosomiasis as risk factor for HIV transmission is more persistent than for STIs, which normally only occur sporadically and have short clinical duration. Both men and women as sexual partners living in *S. haematobium* endemic areas are dually affected by egg-induced genital lesions because both genders are exposed to the same water bodies containing the infective cercarial parasites. Hence, a neglected HIV transmission synergy may exist by which increased HIV-1 RNA shedding in semen due to the MGS-associated lesions in the prostate and seminal vesicles may act in a complementary way to the increased vulnerability to HIV infection in women with FGS due to the coexisting cervico-vaginal lesions. In addition, the effect of an occasional single-dose praziquantel treatment on the infection is commonly transient due to the re-exposure to the *Schistosoma* parasite in the local fresh water bodies. Therefore, repeated praziquantel treatment of the endemic populations by yearly intervals will be required in long-term control strategies targeting children as well as adults.

This study should be followed up by larger randomized controlled trials to assess the effect of praziquantel treatment in reduction of seminal HIV-1 RNA shedding in *S. haematobium* and HIV co-infected men. From an overall HIV control strategy perspective, such trials should also be designed to address the diagnosis and treatment of STIs as a commonly coexisting reproductive health condition in males living in *S. haematobium* endemic areas in sub-Saharan Africa.
